# High-force catch bonds between the *Staphylococcus aureus* surface protein SdrE and complement regulator factor H drive immune evasion

**DOI:** 10.1038/s42003-023-04660-1

**Published:** 2023-03-21

**Authors:** Telmo O. Paiva, Joan A. Geoghegan, Yves F. Dufrêne

**Affiliations:** 1 Louvain Institute of Biomolecular Science and Technology, UCLouvain, Croix du Sud, 4-5, L7.07.07, B-1348 Louvain-la-Neuve, Belgium; 2 Institute of Microbiology and Infection, University of Birmingham, Edgbaston, Birmingham B15 2TT UK

**Keywords:** Pathogens, Single-molecule biophysics

## Abstract

The invasive bacterial pathogen *Staphylococcus aureus* recruits the complement regulatory protein factor H (fH) to its surface to evade the human immune system. Here, we report the identification of an extremely high-force catch bond used by the *S. aureus* surface protein SdrE to efficiently capture fH under mechanical stress. We find that increasing the external force applied to the SdrE-fH complex prolongs the lifetime of the bond at an extraordinary high force, 1,400 pN, above which the bond lifetime decreases as an ordinary slip bond. This catch-bond behavior originates from a variation of the dock, lock and latch interaction, where the SdrE ligand binding domains undergo conformational changes under stress, enabling the formation of long-lived hydrogen bonds with fH. The binding mechanism dissected here represents a potential target for new therapeutics against multidrug-resistant *S. aureus* strains.

## Abstract (web summary)

*S. aureus* uses its surface protein SdrE to form catch bonds with the host regulatory protein factor H and evade the human immune system.

## Introduction

Invasive pathogens infect and replicate in the human host leading to symptomatic diseases. Their success depends on their ability to withstand the complex defense mechanisms of the human host. The complement system, a key component of innate immunity, is composed of a multiplicity of plasma proteins and activated by two specific recognition pathways, the classical and lectin pathways^1^. Complement activation leads to pathogen opsonization, pro-inflammatory anaphylatoxin generation, and assembly of the membrane attack complex to directly lyse Gram-negative bacteria^2–5^.

**Fig. 1:**
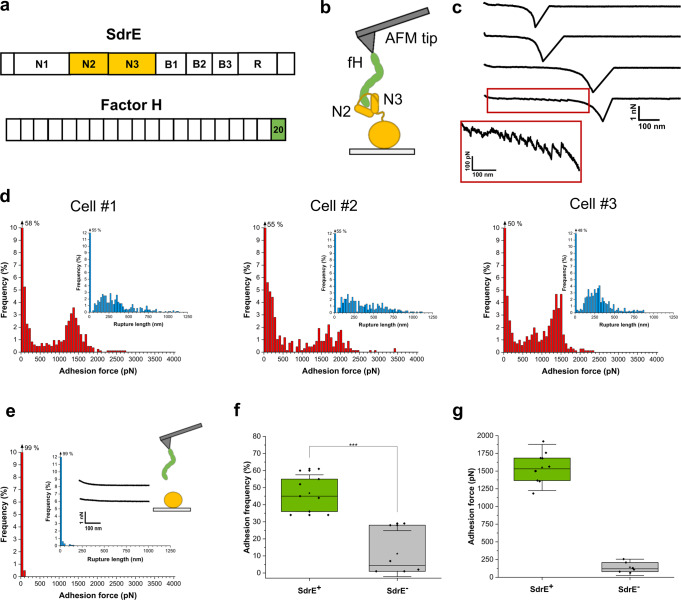
Strength of single SdrE-fH bonds in living bacteria. **a** Schematic representation of the modular structures of SdrE and fH. In SdrE, N1-N3 is the ligand-binding region, B1-B3 the B repeats region and R the serine-aspartate repeat region. The 20 structural units of fH are the CCP (complement-control protein) repeats which are made of β-barrel folds. Here, we used a recombinant fH (amino-acids 860-1231) comprising the 6 terminal CCP modules (CCP15-20). The SdrE N2N3 domains bind a short peptide sequence from fH CCP20, the last fH structural unit. **b** Single-molecule force spectroscopy (SMFS) set-up used in this study. **c** Representative retraction force profiles obtained by recording force-distance curves in PBS between SdrE^(+)^ cells and AFM tips functionalized with fH, documenting either a single adhesion (or “rupture”) peak (three upper curves) or a single adhesion peak preceded by an unfolding force pattern (lower curve). The curve in the lower red box shows an enlargement of the unfolding pattern. **d** Adhesion force and rupture length (insets) histograms generated for three different SdrE^(+)^ cells (*n* = 838, 678, and 706 curves for cell #1, cell #2, and cell #3, respectively). **e** Force data obtained in the same conditions for a SdrE^(−)^ cell (*n* = 1,024 curves). **f** Box plot comparing the adhesion frequency of SdrE^(+)^ and SdrE^(−)^ bacterial strains (*n* = 9 and 6 cells, respectively). ***p-value ≤ 0.001, determined by a two-sample *t*-test in Origin. **g** Box plot of the adhesion force measured for SdrE^(+)^ and SdrE^(−)^ cells (*n* = 9 and 6 cells, respectively). In both **f** and **g**, stars are the mean values, lines the medians, boxes indicate the 25–75% quartiles and the standard deviation (S.D.) is indicated by the whiskers.

Pathogen opsonization by the alternative pathway of the complement system is mediated by activation of C3 convertases, which cleave C3 into C3b fragments that bind covalently to the surface of bacterial cells and act as an opsonin facilitating phagocytosis. To circumvent damage of host cells, down-regulation of the alternative complement pathway is achieved by factor H (fH) (Fig. 1a), a major host regulator of the complement system^5,6^. This glycoprotein, that circulates in human plasma at relatively high concentrations, interacts with surface-bound C3b and cleaves it to its inactive form iC3b.

*Staphylococcus aureus* recruits the complement regulatory protein fH to its surface to inhibit the alternative pathway of complement^7^. This immune evasion strategy relies on the capture of fH by the *S. aureus* cell surface protein SdrE (Fig. 1a)^8^. SdrE binds to the fH C-terminal complement control protein (CCP) modules *via* the recently described ‘close, dock, lock and latch’ (CDLL) mechanism^9^. The ligand binding N2N3 domains of SdrE adopt a closed state in the absence of fH, which undergoes a large conformational change on fH binding. This unique mechanism derived from the classical DLL^10^ allows the pathogen to strongly sequester fH on its surface for complement evasion. Despite the importance of the SdrE-fH interaction for immune evasion, its molecular details are still unknow.

A fascinating aspect of microbiology is to understand how bacteria sense and respond to mechanical cues, such as fluid flow and cell surface contacts^11–13^. In their natural environments, bacterial cells are exposed to physical forces that impact their behavior, and provide feedback to enhance functions such as motility and adhesion. An interesting case is the formation of catch bonds, which unlike classical slip-bonds strengthen under physical stress. The prototypical example is the interaction of the *Escherichia coli* FimH adhesin with mannose, whose strength is increased by fluid flow^14–17^. Despite the importance of catch bonds in firmly stabilizing the interaction of bacterial pathogens with their human hosts, their molecular and atomic details remain elusive, and only few such mechanisms have been identified^13,17^.

Here, we report an extremely high-force catch bond used by a bacterial pathogen as an immune evasion mechanism, focusing on the SdrE-fH interaction. Single-molecule atomic force microscopy (AFM)^18–20^ shows that the SdrE-fH protein complex has a force resilience equivalent to that of a covalent bond. AFM force-clamp experiments reveal that the SdrE-fH bond lifetime first grows with increasing mechanical stress, indicating a catch bond, and then decreases to behave as a slip bond beyond a very high critical force of ~1.4 nN, much larger than for other types of biological catch bonds. Our results imply that mechanical stress triggers structural changes in the adhesin so that the N2N3 domains bind through a DLL (-like) interaction involving long-lived hydrogen bonds. This newly discovered catch bond provides *S. aureus* with a means to efficiently capture circulating fH from plasma and maintain it tightly on the cell surface, highlighting the importance of mechanical stress to enable pathogens to withstand the hostile environment of their host, i.e., the immune system.

## Results

### Strength of the SdrE-fH interaction

We used single-molecule force spectroscopy^20^ (SMFS; Fig. 1) with fH-modified AFM tips to force probe living bacterial cells. To study SdrE in the absence of other staphylococcal cell wall components, we employed a *Lactococcus lactis* strain expressing SdrE (here after SdrE^(+)^ cells)^8^. To ensure single-molecule detection, fH was attached to the tips at low density using a PEG-benzaldehyde linker (Fig. 1b)^20^. Shown in Fig. 1c are representative retraction force profiles obtained between SdrE^(+)^ cells and fH tips. We observed force curves with either a single large adhesion (rupture) peak or with sawtooth patterns made of multiple weaker force peaks. There were also force profiles combining both features, that is, a sawtooth pattern followed by a large adhesion peak. Figure 1d shows adhesion force and rupture length histograms of the large rutpure peaks obtained for three SdrE^(+)^ cells (for more cells, see Supplementary Fig. 1). While rupture distances featured relatively broad distributions, reflecting the various configurations of the elongated fH molecule on the tip and the possible spontaneous formation of dimers, sharp distributions of strong adhesion events were observed, with mean forces of 1355 ± 184 pN (mean ± S.D. on *n* = 375 adhesive curves), 1450 ± 396 pN (*n* = 312) and 1366 ± 159 pN (*n* = 368), for cell #1, cell #2, and cell #3, respectively. Adhesion was mediated by SdrE as it was essentially abrogated in SdrE^(-)^ cells (Fig. 1e and Supplementary Fig. 2), 47% vs 11%, respectively, as shown in Fig. 1f. Here, only very low forces of 141 ± 76 pN were measured (Fig. 1g; mean ± S.D. from a total of 4308 curves from 10 cells), contrasting with the mean adhesion force of the strong peaks, 1551 ± 217 pN (Fig. 1g).

These results indicate that SdrE binds fH extraordinarily tightly. We argue that the ~1.5 nN force is associated with single DLL (or DLL-like) interactions, because: (i) for all cells investigated, adhesion forces featured distributions that were narrow and centered at ~1.5 nN, which strongly supports the idea that single bonds were probed. (ii) Strong forces are in the range of values for the DLL interaction between the structurally related SdrG protein and Fg, both on living bacteria^21^ and on purified adhesins^22–24^. (iii) SdrE has been proposed to bind the fH C-terminus via a DLL-like mechanism^9^.

**Fig. 2:**
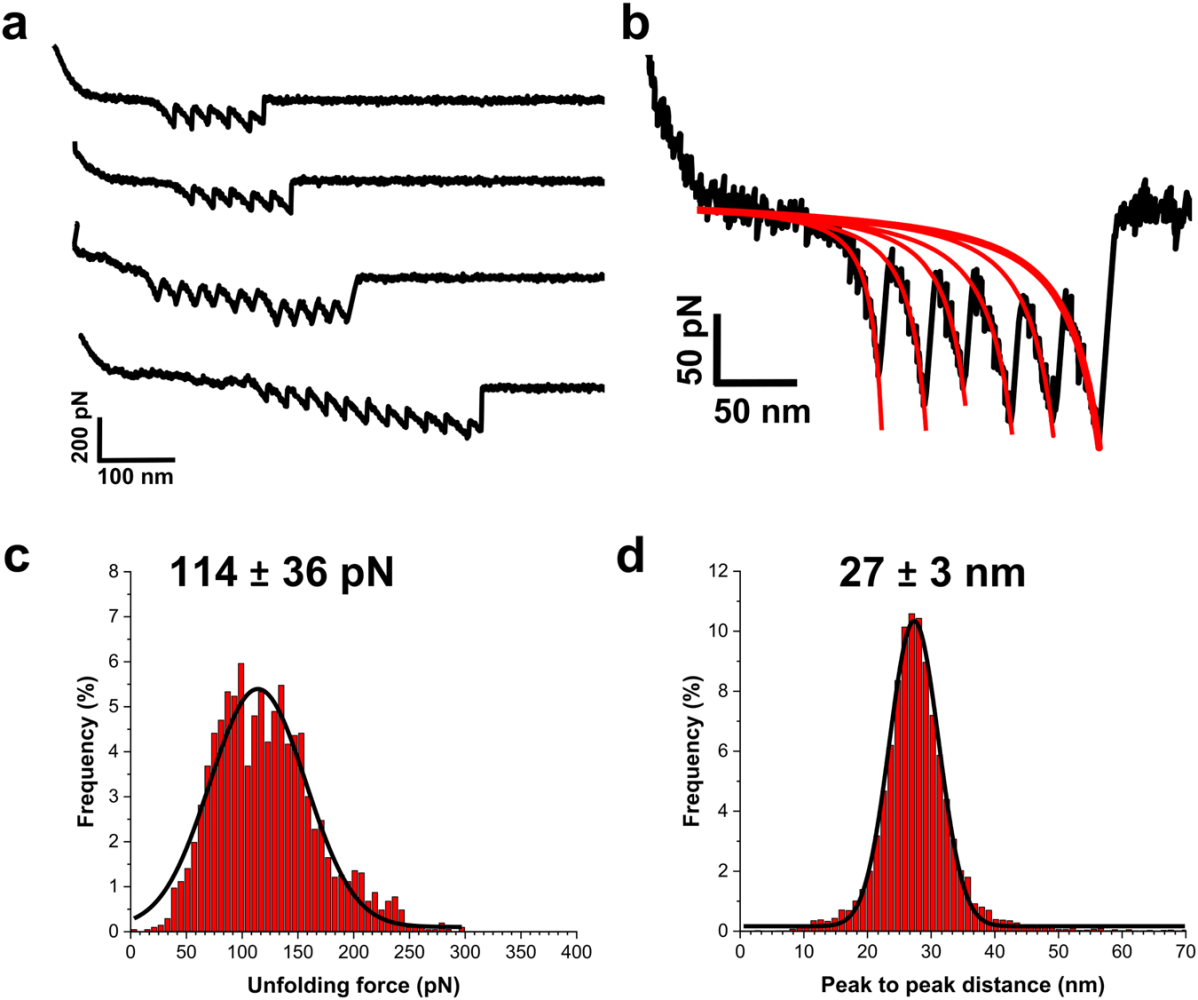
Force-induced unfolding of the fH repeats. **a** Representative retraction profiles obtained by pulling fH tips away from the surface of SdrE^(+)^ cells, featuring the sequential unfolding of individual fH repeats. Two types of multi-peak patterns were observed, reflecting either the unfolding of the six repeats of fH monomers (upper curves) or the twelve repeats of fH dimers (lower curves). **b** Unfolding forces (black line) were well-fitted by the worm-like-chain (WLC) model (red lines), calculated using the equation: $F(x)=\frac{{{{{{{\rm{k}}}}}}}_{{{{{{\rm{B}}}}}}}{{{{{\rm{T}}}}}}}{{l}_{p}}[\frac{1}{4}{(1-\frac{x}{L}+\frac{F}{{{{{{\rm{\varphi }}}}}}})}^{-2}+\frac{x}{L}-\frac{1}{4}]$, where *F* is the unfolding force, *L* the contour length, ${{{{{\rm{\varphi }}}}}}$ the stiffness and ${l}_{p}$ the persistence length. **c** Unfolding force and **d** peak to peak distance histograms resulting from the analysis of the unfolding patterns from three independent SdrE^(+)^ cells (*n* > 2000 and 10,000 unfolding peaks, respectively). Both histograms were well-fitted by a gaussian function (black lines), yielding an average unfolding force of 114 ± 36 pN and an average peak-to-peak distance of 27 ± 3 nm.

The low-force sawtooth patterns that were seen either alone (Fig. 2a) or preceding the large adhesion peaks (Fig. 1c, last curve) result from the sequential unfolding of the structural units of fH, CCP repeats (Fig. 1a), for the following reasons. First, the unfolding forces of the repeats, 114 ± 36 pN (Fig. 2c), compare well with those of modular proteins with β-fold domains, typically in the 100–250 pN range depending on the loading rate^20,25^. Second, about 6 or 12 unfolding peaks were detected in most of the retraction curves, reflecting the unfolding of fH monomers and dimers, as we used a recombinant fH from amino-acid positions 860–1231 made of 6 CCP units. Third, unfolding forces were well-fitted with a worm-like-chain (WLC) model, as shown in Fig. 2b, thus reflecting the unfolding of protein secondary structures^25^, here, the β-barrel folds of fH repeats. Fourth, the peak-to-peak distance, 27 ± 3 nm (*n* > 10,000 unfolding peaks from three cells; Fig. 2d) was close to the length expected for fully unfolded fH repeats, 60 × 0.36−3.3 = 18.3 nm, calculated assuming that one fH repeat comprises 60 amino acids^26^ (each amino-acid contributes 0.36 nm to the length of the unfolded protein; the distance between N-terminus and C-terminus of each fH repeat in its folded state is 3.3 nm, as measured in the crystal structure).

**Fig. 3:**
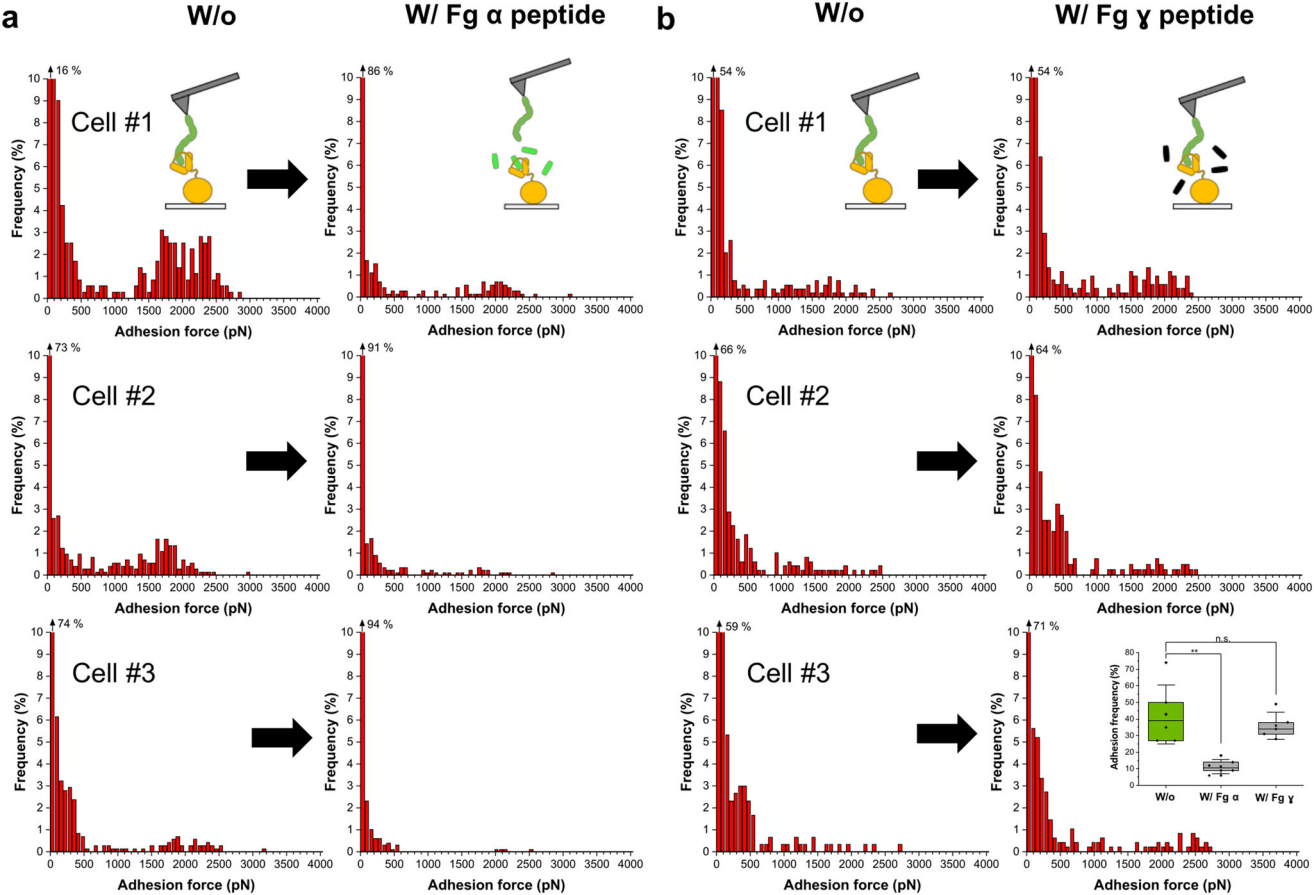
fH-binding by SdrE involves a DLL-like interaction. **a** Adhesion forces obtained by recording force-distance curves in PBS between three different SdrE^(+)^ cells and fH-tips, in the absence or presence of the C-terminal segment (residues 561–575) of the Fg α-chain (0.5 mg mL^−1^). **b** Data obtained for three cells with the C-terminal segment of the Fg γ-chain (0.5 mg mL^−1^). The box plot in inset compares the adhesion frequency in the absence (*n* = 6 cells) and in the presence of either the Fg α-chain (*n* = 6 cells) or the Fg γ-chain (*n* = 5 cells) peptide. **p-value ≤ 0.01; ns not significant, *p*-value > 0.01, determined by a two-sample *t*-test in Origin.

We asked whether it would be possible to inhibit the interaction between SdrE and fH using a putative peptide ligand. The N2N3 domains of SdrE and its allelic variant *S. aureus* bone sialoprotein-binding protein (Bbp)^27^ are structurally related and share 64% amino-acid identity. Bbp recognizes a specific binding site located between residues 561 and 575 of the α-chain of Fg^28^. Therefore, we hypothesized that binding to the α-chain of Fg might interfere with fH-binding by SdrE. To test this, we assessed the ability of a short α-chain peptide to block the adhesive forces of SdrE^(+)^ cells. Figure 3a shows that incubating the cells with this peptide led to a dramatic inhibition of high forces, while leaving low forces essentially unchanged. This finding shows that the residues critical for fH binding overlap with those involved in binding to the Fg α-chain. In contrast a peptide corresponding to the extreme C-terminus of the Fg γ-chain, bound through DLL by *S. aureus* adhesins ClfA and FnBPs, did not block the interaction between SdrE and fH (Fig. 3b).

### Dynamic experiments demonstrate stress-enhanced SdrE-fH binding strengths

**Fig. 4:**
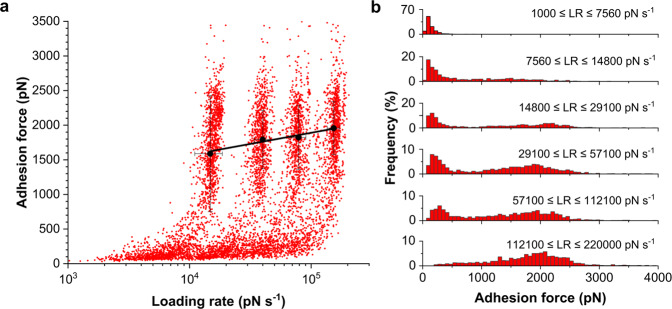
Physical stress enhances the strength of the SdrE-fH interaction. **a** DFS plot showing the adhesion force as a function of the logarithm of the loading rate (*LR*) applied during retraction, while keeping constant the interaction time (50 ms) and the approach speed (1,000 nm s^−1^). Data pooled from five SdrE^(+)^ cells (total of *n* = 5358 adhesive events). Bell-Evans fit (black line) through the most-probable forces showing the expected loading-rate dependency and *x*_*u*_ = 0.03 nm and *k*_off_ = 9.4 ×10^−4^ s^−1^. Error bars represent the standard deviations. **b** Adhesion force histograms as a function of discrete ranges of loading rates documenting a major shift towards higher forces with increasing *LR*s.

We studied the dynamics of the SdrE-fH interaction by measuring the SdrE-fH adhesion forces while varying the loading rate (*LR*, the rate at which force is applied, estimated from the linear slope immediately preceding each unbinding event on the force *vs* time curves) (Fig. 4). As shown in Fig. 4a, the data exhibited a fuzzy distribution with clouds resulting from the different pulling speeds. Fitting these dynamic force spectroscopy (DFS) data with the Bell-Evans model revealed that the adhesion forces increased linearly with the logarithm of the loading rate^20,29^.

Discrete ranges of *LRs* were binned and force distributions were plotted as histograms (Fig. 4b). A population of weak/moderate forces (<500 pN) was observed at low *LRs* but tended to vanish at increased *LRs*. Only strong forces were observed at the highest *LRs* (>1000 pN), meaning tensile loading dramatically strengthens the SdrE-fH interaction. These DFS data lead us to conclude that, as for ClfA, ClfB, and SpsD engaged in DLL (-like) interactions^30–32^, SdrE is a force-sensitive molecular switch that enhances ligand-binding under mechanical tension. This phenomenon is expected to help *S. aureus* to tightly sequester fH at high shear stress, such as encountered in the blood flow.

### A catch bond mechanism activates the capture of fH by SdrE

**Fig. 5:**
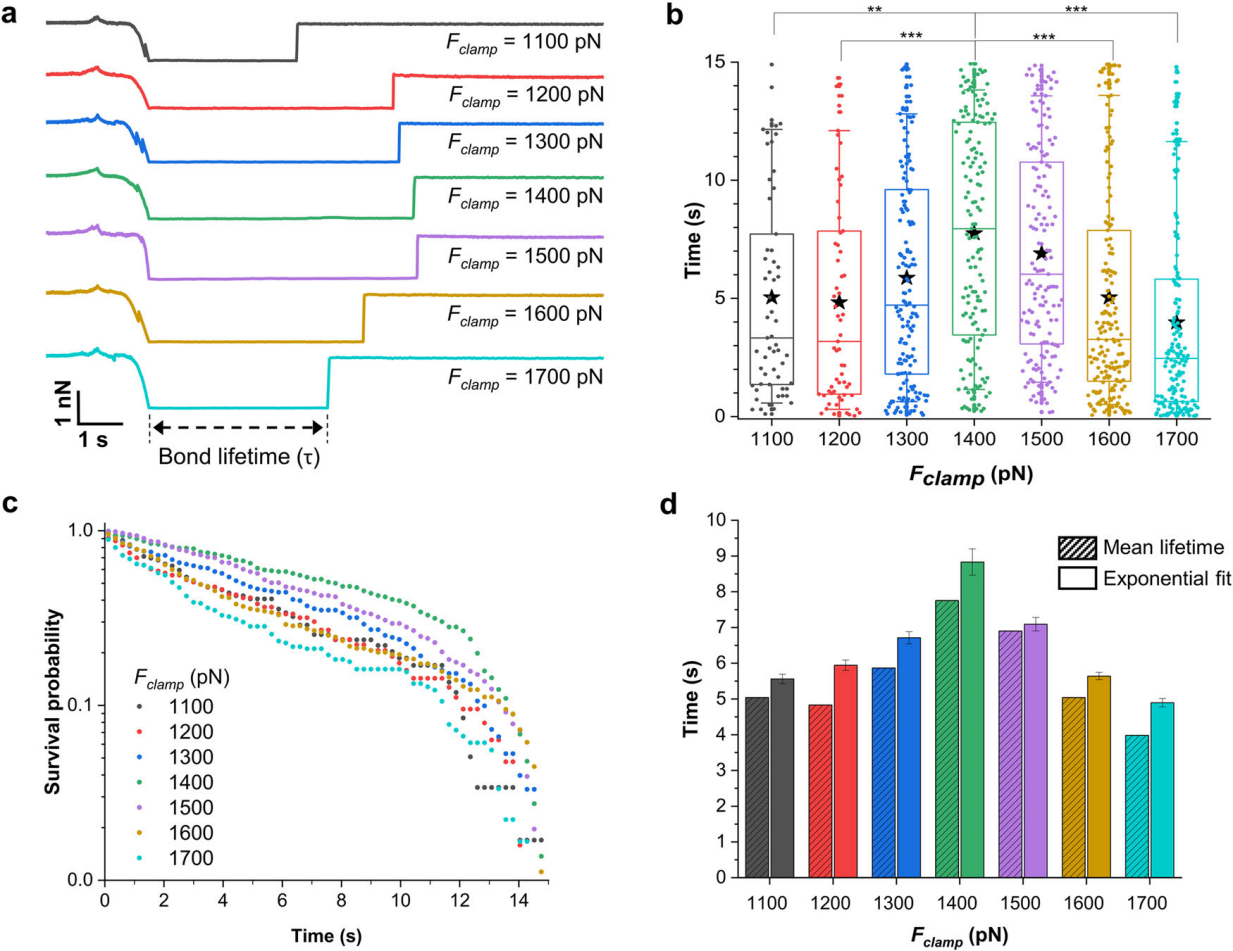
Force-clamp spectroscopy demonstrates that the SdrE-fH interaction involves an extremely high-force catch bond. **a** Representative force-clamp curves (force *vs* time) at increasing *F*_*clamp*_ values (1100, 1200, 1300, 1400, 1500, 1600, and 1700 pN). Each force vs time curve shows different segments, corresponding to the approach, retraction and clamp region, followed by the spontaneous rupture of the SdrE-fH complex. The bond lifetime is given by the constant force regime (clamp segment). **b** Box plot showing the raw lifetimes measured from the force *vs* time curves, for each *F*_*clamp*_ value, at a retraction speed of 1000 nm s^−1^. (A total of 59, 63, 151, 147, 153, 179, and 180 data points are shown for *F*_*clamp*_ = 1100, 1200, 1300, 1400, 1500, 1600, and 1700 pN respectively, collected on 19 SdrE^(+)^ cells in 10 independent experiments). Stars are the mean values, boxes the 25–75% quartiles and whiskers the 10–90% interval. **p-value ≤ 0.01 and ***p-value ≤ 0.001, determined by Dunn-Sidak multiple comparison test. **c** Bond survival probability as a function of time obtained for each *F*_*clamp*_ value. **d** Evidence of catch-slip transition from the representation of mean lifetimes, extracted from the box plot shown in **b**, and bond lifetimes, calculated by fitting the survival plots in **c** with single exponential decays, as a function of *F*_*clamp*_. While the standard deviation of the mean lifetimes must be read in panel **b**, the standard deviation of the bond lifetimes extracted from the exponential fits are shown as error bars, which were calculated from the error of the fits.

We wondered if the dramatic switch in force might result from a catch bond behavior. To provide a direct demonstration of catch-bonding between SdrE and fH, we used single-molecule force-clamp spectroscopy (Fig. 5; 19 cells from 10 independent cultures), in which the bond was clamped at defined loading forces, *F*_*clamp*_, increasing from 1100 pN to 1700 pN (constant retraction speed of 1000 nm s^−1^). An fH-tip was brought into contact with an SdrE^(+)^ cell, pulled apart, and then clamped at a selected *F*_*clamp*_ while registering the time that the adhesin and ligand stay in contact before bond rupture. The bond lifetime (τ) was assessed from the persistent time extracted from the force vs. time curves (Fig. 5a).

Mean lifetimes of 5.0, 4.8, 5.9, 7.7, 6.9, 5.0, and 4.0 s were obtained at *F*_*clamp*_ of 1100, 1200, 1300, 1400, 1500, 1600, and 1700 pN, respectively (Fig. 5b). Hence, τ values increased up to 7.7 s at a critical force of 1400 pN, after which they decreased at higher clamping forces. The τ increase with force indicates a catch-bond, whereas the subsequent decrease reflects an ordinary slip-bond^17^. This biphasic behavior means that the SdrE-fH bond becomes stronger with increasing mechanical load, and then switches back to a weaker state at loads larger than 1400 pN. The same trend was observed for the median lifetime values (Fig. 5b), with even more pronounced differences when force deviates from the critical 1400 pN value. Fitting the mean force-dependent lifetimes with Bell’s model yielded a transition distance of *x*_*u*_ = 0.01 nm in the slip-bond region (>1400 pN), a value close to that estimated by fitting the DFS data through the Bell-Evans model (Fig. 4a, 0.03 nm).

Closer examination of the lifetime data in Fig. 5b shows that at *F*_*clamp*_ of 1400 pN and 1500 pN, the majority of the τ values were equally distributed from 0 to 15 s. However, for other clamping forces, the lifetimes followed an exponential decay, as expected for lifetime measurements. To further dissect this phenomenon, we built up survival plots, Fig. 5c, in which the number of intact bonds is plotted against time^33^ for the different *F*_*clamp*_ values. The τ values were extracted by fitting a single exponential decay to the survival plots (or directly from the negative reciprocal of the slope of the linear fit to the semi-log representation of the survival probability), according to $N\left(t\right)={N}_{0}{e}^{-t/\tau }$ (Supplementary Fig. 3; Supplementary Table 1), yielding bond lifetimes of 5.6 ± 0.1, 5.9 ± 0.1, 6.7 ± 0.2, 8.8 ± 0.4, 7.1 ± 0.2, 5.6 ± 0.1, and 4.9 ± 0.1 s (means ± standard errors of the fits) at clamping forces of 1100, 1200, 1300, 1400, 1500, 1600, and 1700 pN, respectively. The bond lifetimes were similar, yet slightly higher, than those extracted from the raw data (Fig. 5b). Plotting both data sets as a function of force, Fig. 5d, clearly showed the same biphasic relationship with force, confirming the occurrence of a catch-slip transition. The survival plots (Fig. 5c) were well fitted with a single exponential decay for all forces except 1400 pN. Fitting the survival probability *vs* time curves with a double exponential decay (Supplementary Fig. 4 and Supplementary Table 2) revealed that all bond survival probabilities were well-described by a sum of two exponentials, except for 1400 and 1500 pN, therefore confirming that for these two *F*_*clamp*_ values the data sets can be reasonably described by mean τ values extracted from the raw data (Fig. 5b). Lastly, we note that the catch-slip transition identified here occurs at bond lifetimes and critical forces that are larger than for “pure” DLL interactions (2 s and 1100 pN for the interaction between staphylococcal adhesin SpsD and fibrinogen)^32^. In summary, our force-clamp spectroscopy experiments reveal a catch-slip bond transition in SdrE occurring at a very high critical force, probably the strongest ever reported for a biological bond under similar experimental conditions.

## Discussion

**Fig. 6:**
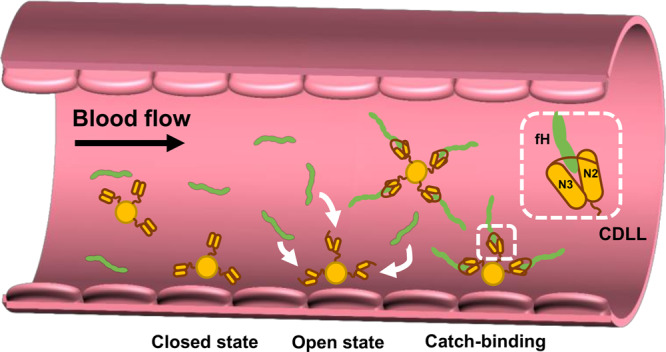
Molecular model of the stress-activated capture of fH by SdrE. Under shear stress of flowing blood, structural alterations in the N2N3 domains of the *S. aureus* cell surface protein SdrE trigger an ultrastrong DLL-like interaction (CDLL) with circulating fH. In vivo, *S. aureus* experiences a wide range of shear stresses throughout the vasculature. While shear rates are low in the veins, they can be very high in arteries, enabling formation of mechanically stable SdrE-fH catch bonds.

Catch bonds play essential functional roles during infection, enabling pathogens to strengthen and stabilize their interactions with their human host targets under mechanical stress^15,17,32,34,35^. While catch bonds have been suggested to be potentially widespread among microbial species, today only few of these have been characterized at the molecular level^34^. By means of single-molecule experiments, we have identified and dissected a novel stress-activated catch bond mechanism between *S. aureus* surface protein SdrE and its ligand complement regulator fH (Fig. 6). The sophisticated stress-dependent stabilization of this protein complex rationalizes at the molecular level the ability of SdrE to efficiently sequester fH under flowing shear stress of the blood, thereby enabling *S. aureus* to withstand the defense mechanisms of the human host.

A first outcome of this study is that SdrE engages in an extraordinarily strong interaction with fH, similar to what has been reported for staphylococcal SdrG, ClfA, ClfB and SpsD DLL (-like) complexes^21–23,30–32^, and that tensile loading induces a dramatic increase in adhesion strength. This indicates that SdrE functions as a force-sensitive molecular switch that enhances ligand-binding under mechanical stress, as observed with catch bonds. We expect this phenomenon to favor efficient sequestration of fH at the cell surface under shear stress associated with blood flow.

Our most remarkable finding is that the SdrE-fH interaction exhibits a catch-slip transition. The bond lifetime increases with force up to ~8 s, and then decreases beyond a critical force of ~1.4 nN to behave as an ordinary slip bond. Surprisingly, the bond lifetime and critical force of the SdrE-fH catch-slip transition are substantially larger than those of DLL interactions^32,34^. We argue that this difference results from the CDLL mechanism recently described for the SdrE-fH interaction^9^, where the SdrE N2N3 domains are in a closed state in the absence of fH, with the binding pocket inaccessible, but undergo a large conformational change upon ligand binding. In summary, our results imply that, under mechanical tension, force-dependent CDLL complexes are more stable than classical DLL ones, suggesting that *S. aureus* may have evolved this catch bond mechanism to strongly capture and sequester fH for efficient immune evasion.

The mechanical stresses applied here are encountered in vivo, as bacterial cells are exposed to various shear stresses in flowing blood (Fig. 6). While shear rates are low in the veins, about 10 s^−1^, they can exceed 2000 s^−1^ in arterioles, and reach values of 40,000 s^−1^ in atherosclerotic arteries^36^. This means that bacteria experience *LRs* under physiological conditions that can exceed 10^5^ pN s^−1^, estimated using the Stokes equation^13^ and considering a blood flow velocity of $\sim \!1$ mm s^−1^ and the radius of the bacterial cells as $\sim$ 0.5 µm. Thus, the range of *LRs* that we tested is biologically-relevant^35^, and enables formation of mechanically stable SdrE-fH catch bonds.

Several models have been proposed to explain counterintuitive shear-enhanced catch bond behaviors. Some of these are simple and based on mechanical concepts, e.g. sliding-rebinding model^37^, while others are more complex, inspired by the biological concept of allostery like the one-state^38^ and two-state catch bond models^39–42^ implying a conformational change of the receptor (SdrE). Interestingly, our force-clamp results do not follow any of these models proposed before, since we observe that for force values close to the critical force of 1400 pN, the survival plots cannot be well-fitted with either single or double exponential decay equations. However, as we deviate from this *F*_*clamp*_ value, the lifetime data seem to follow exponential decay trends, with either a single or multiple fast and slow components. This observation is not surprising, since all these models were proposed to describe transitions that occur under low tensile force, while here a very high-force regime comes into play.

Although high-resolution structures are needed to provide full atomic details on the CDLL catch bond, it is tempting to speculate on the molecular and atomic origin of this bond based on recent simulation studies. The high mechanical stability of the DLL complex between SdrG and fibrinogen results from an intricate hydrogen bond network between the ligand peptide backbone and the adhesin^22^. Molecular dynamics (MD) simulations of this DLL interaction revealed that the target peptide confined in a screw-like manner in the binding pocket of SdrG requires the simultaneous rupture of numerous hydrogen bonds in a cooperative shear geometry, yielding an extreme mechanical strength and highly stable complexes. Another in silico single-molecule study recently demonstrated that SdrG uses a catch bond mechanism to enhance protein complex stability with increasing mechanical stress^43^. While allowing for thermal dissociation in a low-force regime^44^, an entirely different mechanical dissociation path emerged in a high-force regime, revealing a complex mechanism that does not depend on the peptide amino acid sequence. The key amino acid contacts that describe the mechanics of this protein complex were identified, revealing differences in dynamics that hinder thermal dissociation and establish the mechanical dissociation path. We postulate that the higher bond lifetime and critical force associated with the SdrE-fH catch-slip transition results from the CDLL mechanism^9^. Under mechanical stress, fH binding by SdrE involves large conformational changes in the N2N3 domains, and key amino acid contacts that may substantially differ from those of DLL interactions.

The question arises whether and how the fH repeat unfolding events contribute to the mechanical stability of the SdrE-fH complex. We suggest that the adhesive properties of fH are promoted by a switch towards an extended, unfolded conformation under flow, exposing the proper sites for optimal binding to SdrE, a model consistent with the notion that the CDLL interaction involves a conformational change in fH before binding to SdrE^9^. This putative force-dependent function is reminiscent of an essential mechanosensitive multimeric glycoprotein found in the blood, von Willebrand factor (vWF). Upon secretion into the blood, globular vWF becomes extended under flow and responds to mechanical forces, such as shear stress in flowing blood, which is critical for the protein biological functions^45–47^. It has previously been reported that fH captured by the *Streptococcus pneumoniae* surface protein PspC is held in a conformation that exposes a cryptic binding site so that it has a higher affinity for C3b and a greater ability to accelerate decay of the C3 convertase C3bBb^48^. While fH captured by SdrE is active, future studies should investigate if SdrE-captured fH has an enhanced function.

During invasion, bacterial pathogens have to withstand the complex defense mechanisms of the human host. The stress-dependent SdrE-fH catch bond identified here is of biological significance as it represents a competitive advantage to enable *S. aureus* to counteract the antibacterial effects of the innate immune system by capturing and sequestering fH under flowing shear stress of the blood. This evasion strategy allows the bacteria to exploit the function of fH as a negative regulator of complement, reducing opsonisation with C3 fragments which protects the bacteria from phagocytic killing by polymorphonuclear leukocytes^8^. The catch-binding mechanism represents a potential target for new therapeutics that would inhibit fH capture and evasion of complement, thus offering an alternative to conventional antibiotics that are no longer active against multi-drug resistant *S. aureus* strains.

## Methods

### Bacterial strains and growth conditions

*Lactococcus lactis* was used for heterologous expression of *Staphylococcus aureus* SdrE^8^. *The L. lactis* strain MG1363 carrying the pKS80 plasmid containing the WT SdrE gene (pKS80::SdrE)^49^ and the *L. lactis* strain transformed with the empty pKS80 plasmid^49^ were cultured in Brain Heart Infusion (BHI) plates containing erythromycin (10 µg mL^−1^) overnight, at 30 °C. A single colony was selected and transferred to 10 mL of BHI broth supplemented with the same antibiotic and incubated at 30 °C for 16–20 h. Cultures were harvested by 5 min centrifugation at 2000×*g*, washed twice with PBS and resuspended in the same buffer. The bacterial suspension was finally diluted 100 times in PBS and 200 µL of it were deposited on a polystyrene Petri dish and allowed to adhere for 20 min. The Petri dish was washed twice and filled with 2 mL of PBS prior to the AFM experiments.

### Functionalization of AFM tips

MSCT-C cantilevers (Bruker) were functionalized with fH through NHS-PEG_24_-Ph-aldheyde linkers^50,51^ (Broadpharm). The cantilevers were first immersed in chloroform for 10 min, thoroughly rinsed with ethanol, gently dried with nitrogen and further cleaned in a UV-ozone chamber for 15 min. The freshly cleaned cantilevers were then amino-functionalized in gas phase, in a desiccator, where two small containers with 30 µL of 3-aminopropyltriethoxysilane (APTES) and 10 µL of triethylamine were placed. After 2 h incubation, the desiccator was flushed with argon and the cantilevers allowed to curate for at least 2 days. The silanized cantilevers were subsequently incubated with 0.5 mL of chloroform containing 3.3 mg of the NHS-PEG_24_-Ph-aldheyde linker, to which 30 µL of triethylamine were added. After 2 h incubation, the cantilevers were washed 3 times with chloroform and dried with nitrogen. A 30 µL drop of a 1 mg mL^−1^ factor H (CompTech) solution in PBS was pipetted on the cantilevers previously placed on a Petri dish surface covered with parafilm. 1 µL of 1 M freshly prepared solution of sodium cyanoborohydride was added to the drop and left to incubate for 1 h. 5 µL of 1 M ethanolamine solution, pH 8.0, were subsequently added to the drop and left to react for 10 min, blocking the aldehyde groups, exposed at the surface of the cantilevers, that didn’t react. Finally, the cantilevers were washed 3 times in PBS and stored in it until they were used.

### Single-molecule force spectroscopy

Single-molecule force spectroscopy (SMFS)^20^ experiments were performed in PBS, at 20 °C, using a JPK NanoWizard®4 Nanoscience AFM. fH-functionalized cantilevers were calibrated by the thermal noise method, yielding spring constants in the 0.02–0.03 N m^−1^ range. Force-distance curves were recorded on the top of single bacterial cells (500 × 500 nm areas), in force mapping mode (32 × 32 force curves) using constant approach and retraction speeds of 1 µm s^−1^, 1.4 µm ramp length and applying a force of 250 pN. The contact time was 50 ms. Dynamic force spectroscopy experiments were performed by changing the retraction speed from 1 to 2.5, 5 and 10 µm s^−1^. All force curves were analyzed using JPK Data Processing software (version 6.1.172), considering the last adhesion peak, which was fit using the worm-like chain model of polymer extension^52^. For blocking assays, we used either α-chain fibrinogen peptide 561-575 (SKQFTSSTSYNRGDS, Genscript) or 17-mer ɣ-chain fibrinogen peptide (GEGQQHHLGGAKQAGDV, Genscript) at a final concentration of 0.5 mg mL^−1^. Force maps were recorded on the top of the same cell before and after peptide injection.

### Force-clamp spectroscopy

Force-clamp experiments were performed in force mapping mode, recording 16 × 16 force curves on 500 nm × 500 nm areas on the top of single bacterial cells expressing SdrE. For each force curve, MSCT AFM cantilevers functionalized with fH, through the PEG-aldheyde method described above, were brought in contact with the bacterial cell, at 1 µm s^−1^ and applying a force of 250 pN. After contacting its surface, the cantilever was retracted at 1 µm s^−1^ and stopped at a target force, *F*_*clamp*_, at which the SdrE-fH bond was clamped for a maximum time of 15 s. This is possible due to a feedback loop that continuously adjusts the tip-sample distance to maintain the force constant. Lifetimes were extracted from the persistent time of the bond, read on the force vs time curves from the moment the bond was clamped until its spontaneous rupture. To avoid possible artifacts due to the regularization times of the feedback loop, only lifetimes above 20 ms were considered. Raw lifetimes were then averaged to obtain mean lifetimes at *F*_*clamp*_ values of 1100, 1200, 1300, 1400, 1500, 1600, and 1700 pN. Bond survival probabilities (SP) were calculated according to: SP = (100–*fc*)/100, where *fc* are the cumulative frequencies obtained after distributing the lifetimes measured at a given force into time bins. Bond lifetimes were extracted from the survival plots through either single or double-exponential decays.

### Statistics and reproducibility

Statistical analyses were performed with Origin software (OriginPro 2021). Statistical differences between binding frequencies obtained for SdrE^(+)^ and SdrE^(−)^ strains were calculated applying a Student’s *t* test. A Dunn-Sidak multiple comparison test was employed to assess the differences between lifetime values obtained for different *F*_*clamp*_ values. *P*-values are reported in the figure captions. Sample sizes and replicates are reported in the figure captions as well as along the text. Experiments were repeated at least twice.

### Reporting summary

Further information on research design is available in the Nature Portfolio Reporting Summary linked to this article.

## Supplementary information

Supplementary Information

Description of Additional Supplementary Files

Supplementary Data

Reporting summary

## Data Availability

All data generated or analyzed during this study are provided in Supplementary Data. All other relevant data are available from the corresponding author on reasonable request.
