# Supplementary material for: High-force catch bonds between the Staphylococcus aureus surface protein SdrE and complement regulator factor H drive immune evasion
Source: Commun Biol. 2023 Mar 21;6:302. doi: 10.1038/s42003-023-04660-1 (PMC10030832; doi:10.1038/s42003-023-04660-1)
Supplement: Supplementary file 1 — Supplementary Information [file 42003_2023_4660_MOESM1_ESM.pdf]

Supplementary information

High-force catch bonds between the *Staphylococcus aureus* surface protein SdrE and complement regulator factor H drive immune evasion

Telmo O. Paiva[1], Joan A. Geoghegan[2*], and Yves F. Dufrêne[1*]

***Corresponding authors:**

Yves F. Dufrêne: yves.dufrene@uclouvain.be

Joan A. Geoghegan: j.geoghegan@bham.ac.uk

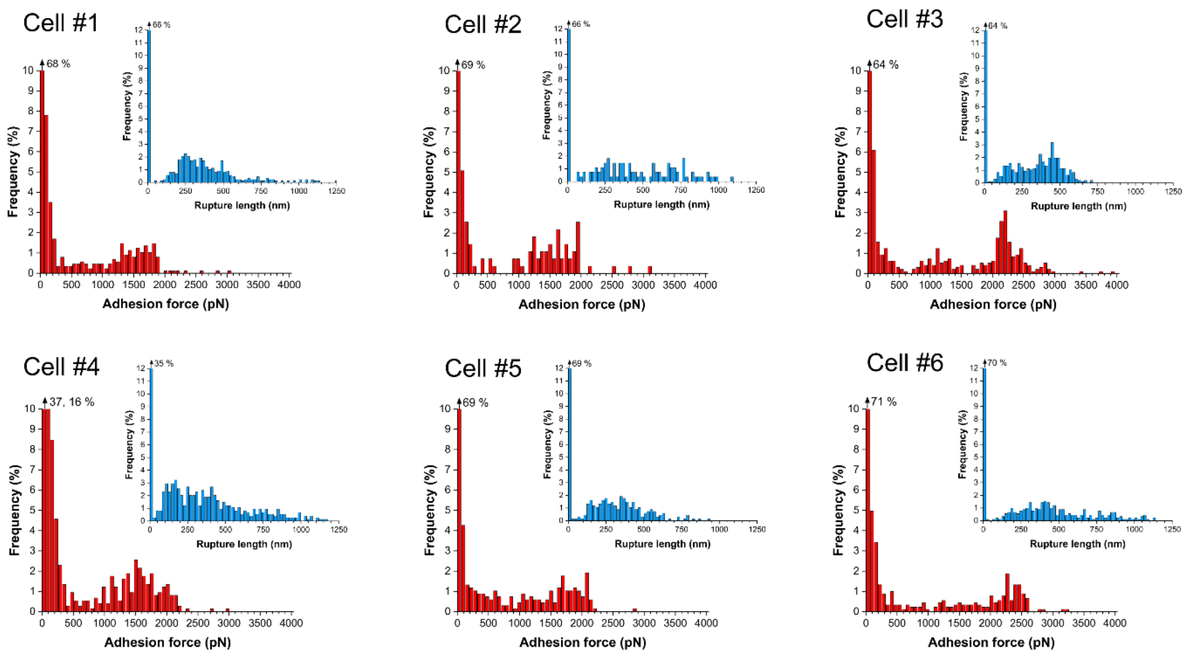

Supplementary Figure 1. Single molecule force spectroscopy of the SdrE-fH interaction. Adhesion force and rupture length histograms (inset) generated for six different SdrE$^{(+)}$ cells (n = 885, 276, 970, 744, 681 and 905 curves for cell #1, cell #2 and cell #3, cell #4, cell #5 and cell #6, respectively). Experiments were performed at room temperature, in PBS and applying a retraction speed of 1,000 nm.s^{-1}.

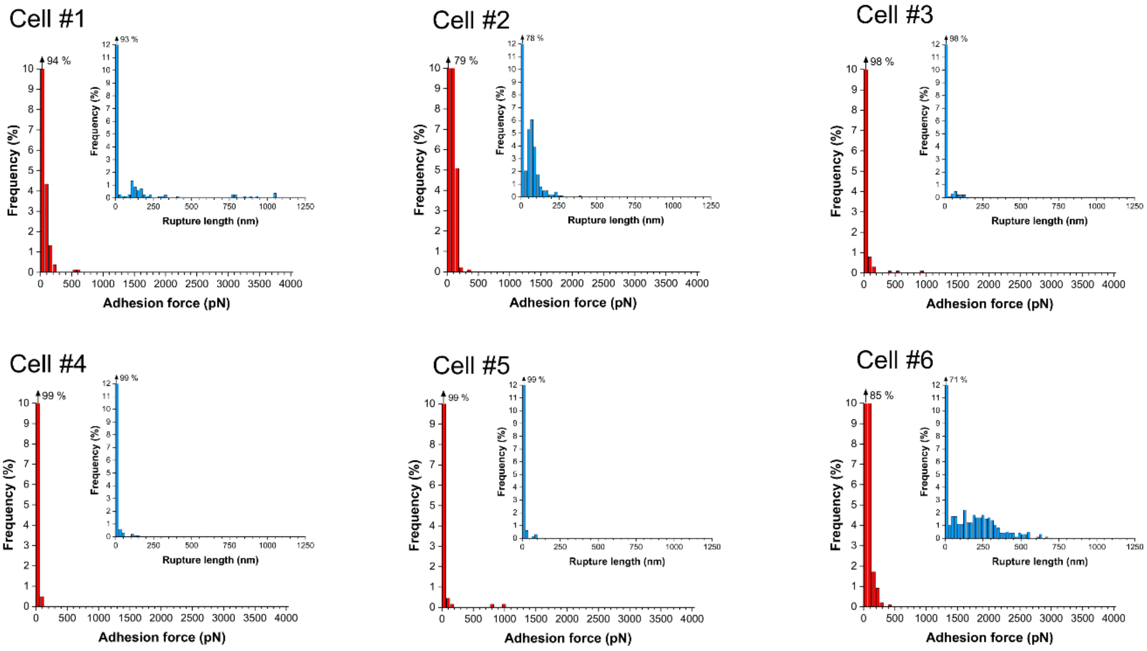

Supplementary Figure 2. Single molecule force spectroscopy of the SdrE-fH interaction. Adhesion force and rupture length histograms (inset) generated for six different SdrE$^{(-)}$ cells (n = 831, 1024, 1024, 1024, 673 and 996 curves for cell #1, cell #2 and cell #3, cell #4, cell #5 and cell #6, respectively). Experiments were performed at room temperature, in PBS and applying a retraction speed of 1,000 nm.s^{-1}.

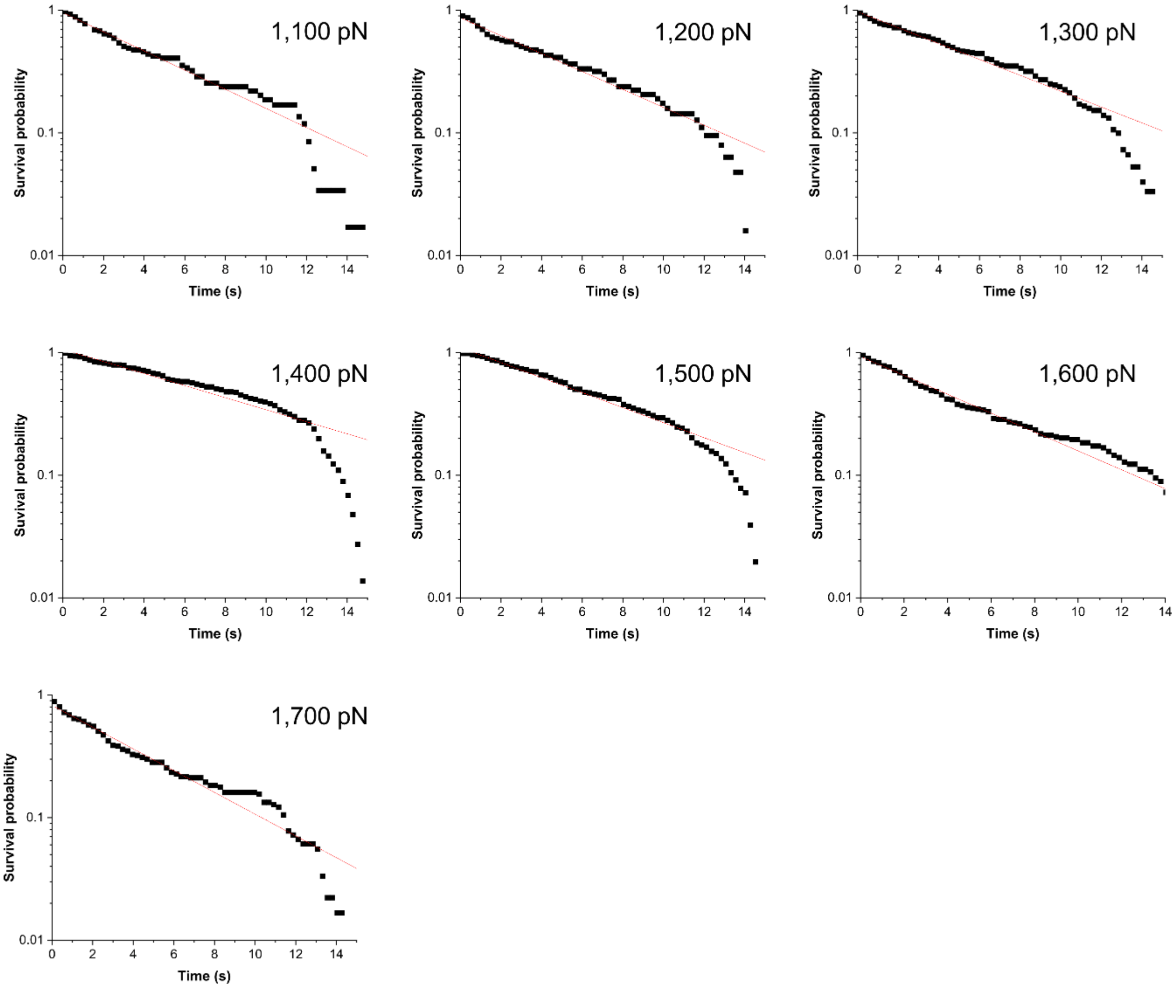

Supplementary Figure 3. Single exponential decay fits of the bond survival probabilities for the SdrE-fH interaction at different clamping forces. In black are represented the bond survival probabilities as a function of time. Single exponential fits are represented in red.

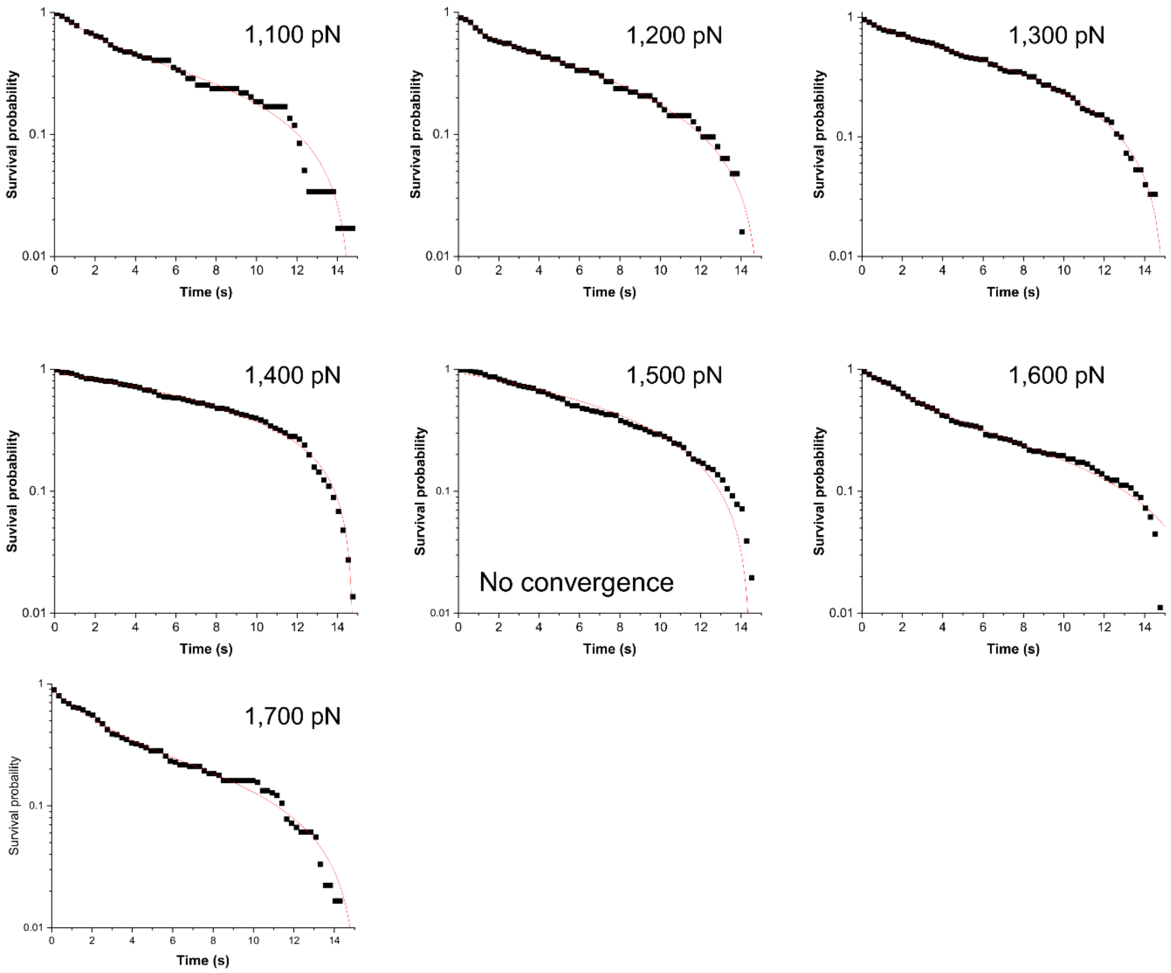

Supplementary Figure 4. Double exponential decay fits of the bond survival probabilities for the SdrE-fH interaction at different clamping forces. In black are represented the bond survival probabilities as a function of time. Double exponential fits are represented in red.

Supplementary Table 1. Fitting parameters obtained by fitting the survival plots at different clamping forces by a single exponential decay. The bond lifetime is given by τ_1.

F_{clamp} (pN)	Max. number of iterations	Convergence	τ_1 (s)	A1	X^2	R^2
**1,100**	400	Yes	5.560 ± 0.133	0.957 ± 0.015	0.001	0.979
**1,200**	400	Yes	5.945 ± 0.145	0.869 ± 0.014	0.001	0.978
**1,300**	400	Yes	6.714 ± 0.170	0.971 ± 0.016	0.002	0.976
**1,400**	400	Yes	8.832 ± 0.367	1.062 ± 0.026	0.005	0.930
**1,500**	400	Yes	7.092 ± 0.187	1.100 ± 0.018	0.002	0.973
**1,600**	400	Yes	5.640 ± 0.102	0.930 ± 0.011	7.204×10^{-4}	0.987
**1,700**	400	Yes	4.895 ± 0.116	0.823 ± 0.013	8.891×10^{-4}	0.980

Supplementary Table 2. Fitting parameters obtained by fitting the survival plots at different clamping forces by a double exponential decay. The bond lifetime is described by two different components, τ_1 and τ_2.

F_{clamp} (pN)	Max. number of iterations	Convergence	τ_1 (s)	A1	τ_2 (s)	A2	X^2	R^2
**1,100**	400	Yes	1.857 ± 0.229	0.476 ± 0.033	1979.148 ± 46116.542	74.428 ± 1723.461	4.453×10^{-4}	0.999
**1,200**	400	Yes	0.932 ± 0.121	0.318 ± 0.020	23.427 ± 5.302	1.396 ± 0.194	1.676×10^{-4}	0.999
**1,300**	400	Yes	1.563 ± 0.359	0.193 ± 0.031	43.016 ± 20.750	2.609 ± 0.949	1.182×10^{-4}	0.998
**1,400**	400	Yes	-1.582 ± 1.163	-9.057E-6 ± 6.762×10^{-5}	14610.338 ± 4852740.385	-830.150 ± 275812.294	3.809×10^{-4}	0.995
**1,500**	1000	No. Overparameterization	-	-	-	-	-	-
**1,600**	400	Yes	2.538 ± 0.179	0.607 ± 0.021	3534.427 ± 75737.194	82.946 ± 1771.369	2.574×10^{-4}	0.999
**1,700**	400	Yes	1.974 ± 0.133	0.543 ± 0.016	5564.385 ± 49234.961	135.289 ± 1194.199	2.791×10^{-4}	0.999
